# Assisted Protection Headphone Proposal to Prevent Chronic Exposure to Percussion Instruments on Musicians

**DOI:** 10.1155/2018/9672185

**Published:** 2018-02-08

**Authors:** Lorena Parra, Marta Torres, Jaime Lloret, Agustín Campos, Ignacio Bosh

**Affiliations:** ^1^Instituto de Investigación para la Gestión Integrada de Zonas Costeras (IGIC), Universitat Politècnica de València, Valencia, Spain; ^2^ENT Unit, Surgical Department, Valencia Medical School, University General Hospital of Valencia, Valencia, Spain; ^3^Institute of Telecommunications and Multimedia Applications (iTEAM), Universitat Politècnica de València, Camino Vera n/n, 46022 Valencia, Spain

## Abstract

The effects of chronic exposure to high sound pressure levels (SPLs) are widely studied in the industry environment. However, the way that SPLs affect music students has not been thoroughly examined. In this paper, we study the SPL exposure of batucada students and we propose an assisted protection headphone as a part of e-health system. We measured the SPL reached during a regular percussion class. Pure-tone audiometries were performed to a set of percussion students. The gathered data were statistically analyzed. The assisted protection headphones and their operation are detailed and tested during a regular class. Our results show that 35% of the musicians present with a noise-induced hearing loss, considered as two frequencies with hearing loss of 25 dB or more or one frequency with a hearing loss of 30 dB or more. Our data also shows that those students that do not use any protection have a greater hearing loss. However, the students that use protection headphones are also suffering hearing loss. There might be a problem in the way that musicians are using the protection headphones. Our assisted protection headphones as a part of e-health measures can diminish the negative effects of percussion instruments for musicians.

## 1. Introduction

The effects of chronic exposure to high sound pressure levels (SPLs) are widely studied. It is well known that the occupational exposure to high SPL produces noise-induced hearing loss (NIHL) [1]. NIHL is considered, after presbycusis, the second most common form of sensorineural hearing deficit. In order to identify a specific noise, the intensity and the frequency must be identified. The intensity, commonly known as SPL, is expressed in dB, while the frequency is expressed in Hz. There are different ways to measure the sound pressure level; in this paper, we will work with A-weighting. The human hearing ability changes at different frequencies. Noise can cause a permanent hearing loss in individuals with chronic exposure at 85 dB during eight hours per day [1]. Increasing intensity reduces the considered exposure period.

The exposure to high SPLs has been widely studied in the work environment. Nevertheless, in the leisure environments, it has been less studied. Recent studies have focused on the effect of personal hearing devices or headphones [2, 3]. However, the effect of SPLs registered while playing music has not been thoroughly studied. Specifically, different music styles can reach different SPLs. Typical SPL between classical musicians varies between 80 dB and 90 dB. Drum players, however, are submitted to higher sound levels with peak values up to 125 dB [4, 5]. We have focused this research on members of a drum school to study NIHL along with the prevalence of accompanying hearing symptoms. A further objective was to assess related factors such as the type of hearing protection employed. In the school, they have different protection equipment as earplugs or soundproof protection headphones. Nevertheless, not all the students use them or take care of adjusting them correctly.

The e-health systems are becoming more and more useful, and many authors are developing applications for monitoring different issues [6]. Some examples are focused to gather data from patients in order to prevent or monitor some diseases [7, 8] or for people with special needs as elderly people [9] or for weight control [10]. On the other hand, the wireless sensor network can gather data form biomedical sensors and environmental sensors. This combination of possibilities is needed from many bridging and routing protocols as other wireless sensor networks (WSN) [11, 12]. The use of internet of things (IoT) has been employed in ambient assisted living in several cases [13]. Moreover, the minimization of energy consumption is one of the main topics to deal with it [14].

The aim of this paper is to (i) study the effects of SPL reached while playing batucada music in the hearing capability of the musicians, (ii) design an assisted protection headphone, and (iii) evaluate its function. To evaluate the effects of SPL, first, we perform some records during a regular batucada class in “Borumbaia drum school” in Valencia. For the measurements, sound pressure meters of Brüel & Kjær were used. Specifically, the models 2250 and 2270 were employed. The advanced FFT analysis and the frequency analysis modules were employed. Then, pure-tone audiometries are done to the students and teachers in the ear, nose, and throat department of the University General Hospital of Valencia. We will analyze the response of people to frequencies from 100 Hz to 8000 Hz. The use of protections, the period of exposition, and the played musical instrument are other studied variables. Moreover, we will try to relate the frequencies where there are NIHL in the students with the frequencies where the sound level meters registered the maximum SPL. In addition, the design of an assisted protection headphone is shown. This protection headphone will be a part of e-health system for the prevention of NIHL for musicians. It will gather the SPL received by the musicians and send this information to the medical center after each class. The motivation to design these headphones is that many musicians that are using the headphones are reporting hearing loss. For this reason, we suspect that some musicians may not be using them properly. The assisted protection headphone is composed by on-sound sensor in the internal part on each side. The headphone has a vibrating element that alerts to the user that it is not well adjusted. Moreover, the designed protocol for generating individual or collective alarms when the SPL detected is above the established threshold is presented. Finally, the assisted protection headphones are tested during a regular class to evaluate its performance.

The rest of this paper is structured as follows, Section 2 presents the related work. The details of the material and methods employed in this study are shown in Section 3.  Section 4 details the design of the assisted protection headphones and its operation.  Section 5 shows the results of the performed study, the gathered data during batucada class, and the audiometries, and the test of the assisted protection headphones are presented there. Finally, conclusions and future work are presented in Section 6.

## 2. Related Work

In this section, we will review the current knowledge about sound exposure in professional and nonprofessional musicians. We will look for differences and similarities with our present study and break down the auditory aspects studied both objective and subjective (hearing symptoms).

Starting with nonprofessional musicians, Schmuziger et al. [15] compared 42 leisure pop/rock musicians with a control group of 20 members. They rehearsed 5 years minimum for at least 2 hours per week. They found a 26% prevalence of hyperacusis and 17% of tinnitus. Pure-tone audiometry shows a statistically significant difference in the frequency range of 3 to 8 kHz between both groups. The hearing thresholds between the musicians using protection and those who had never used it were also statistically significant.

Pawlaczyk-Łuszczyńska et al. [16] studied a group of 168 academic orchestra students with 6.5% of percussionists (*n* = 11). They gathered information about SPL during individual and group playing. The information was obtained with personal sound exposure meters (4436 and 4443 of Brüel & Kjær). The difference of SPL between instruments was statistically significant, with the percussionists taking the worst part. Furthermore, the hearing threshold at 4 kHz to 8 kHz was not statistically significant between the students and a 67-people control group. They compared their data with the International Standard Organization standard ISO 1999 : 2013 finding poorer hearing results in their sample at 6 kHz and better results at 3 kHz and 4 kHz. Symptoms as tinnitus and hyperacusis were also found in greater proportions in the musical student group compared with the group that was not exposed to loud sounds (11.3% versus 4.5% in tinnitus and 36.3% versus 11.9% in hyperacusis).

Størmer et al. [4] studied a sample of 111 professional rock musicians, finding hearing loss in 37.8% of them, and the worst frequency notch was found at 6 kHz. Only 2.5% of the control group gathered the criteria to be classified as hearing loss. No significant association between an instrument group and hearing loss was uncovered. However, drum players were the larger group among the population with hearing loss, representing 24%. There were statistically significant auditory differences between musicians using hearing protections of any kind and those who have never or infrequently used them. In this study, percussionists had better auditory outcomes than bassists or guitarists. Almost 20% of the musicians had tinnitus.

Rock professional musicians, along with pop and jazz musicians, were studied by Halevi-Katz et al. [5]. They studied for exposure to music and NIHL. They analyzed the people with a questionnaire and a pure-tone audiometry from 1 to 8 kHz. Their results concluded that hearing thresholds were significantly worse in percussionists compared to other musicians. However, the link between hyperacusis and hearing thresholds was not statistically significant while in the left ear, tinnitus and hearing loss were correlated significantly.

Patil et al. [17] did not find the statistically significant differential risk of hearing loss between different instrumental groups with an odds ratio for hearing loss in percussionists of 1.83 compared with woodwind instrumentalists. They performed pure-tone audiometry along with an interview with professional musicians and at control group composed by nonmusician soldiers. There was no difference between groups because the control group was also exposed to occupational noise.

Schink et al. [18] studied a historical cohort of 2227 musicians, 238 of them suffered hearing loss in a four-year observation period. The adjusted hazard ratio of musicians was 3.61 for NIHL and 1.45 for hearing loss; they did not specify the instruments played by the musicians studied. Analyzing orchestra musicians' sound exposure, Rodrigues et al. [19] found that percussionists were exposed to the high peak noise levels of 135 dB while brass players suffered the highest mean exposure 87.7 ± 2.97 dB. However, they did not measure the effects on hearing thresholds or subjective symptoms.

The presented papers show the current knowledge about the NIHL in the musicians. Some authors point that musicians that play different instruments present different NIHL. In some studies [4, 5, 7, 19], the percussionists are the group that presents highest NIHL. However, there is no study that evaluates the NIHL in different percussionists, specifically in percussionists that play batucada. Moreover, in our paper, we evaluate the benefits of using different protection measures.

## 3. Material and Methods

In this section, the material and methods used are detailed. Firstly, the equipment used to gather data about the sound levels in the batucada class is shown. Secondly, the medical equipment employed in the audiometries is presented. The data related to the studied population and the characteristics of the class is detailed. Finally, the musical instruments and the protection measures that the school offers are shown.

### 3.1. Equipment Used to Record the Sound Levels

**Figure 1:**
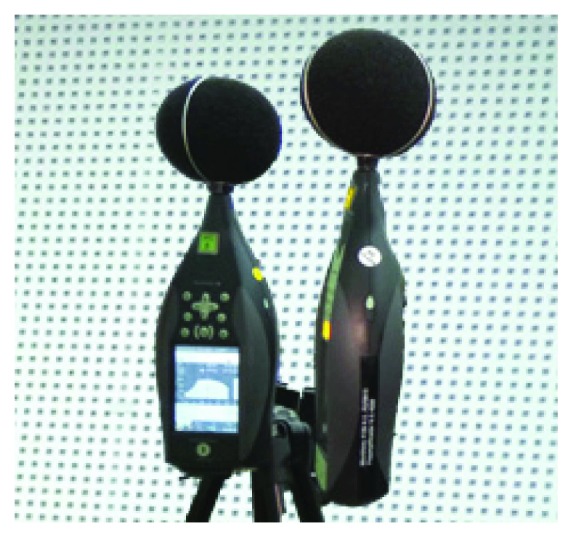
Employed sound level meters.

In this subsection, the employed equipment to perform the records is shown. Two different sound level meters were used. Both of them were manufactured by Brüel & Kjær. The used models are the 2250 and the 2270. The windscreen was used in all the measures. The reason why we use the windscreen is that in future work, we want to record in the streets during a live performance and not only in the classrooms. In the streets, we will use the windscreen. As we want to maintain the same equipment in order compare the records in both cases, we will use the windscreen in the rooms even that it is not necessary. Both devices and the windscreen can be seen in Figure 1.

One of the sound level meters is gathering data related to the advanced FFT analysis. The analysis is performed from 50 Hz to 20,000 Hz with increments of 50 Hz. Three different records were done during different moments of the class. The records have an average time of 35 s. For each record, it is possible to obtain the mean data and the maximum data for each studied frequency. Data were obtained using A-weighting.

The other sound level meter is gathering data related to the frequency analysis. It provides real-time analysis of the 1/1- and 1/3-octave filter band. The scanned frequency goes from 12.5 Hz to 20,000 Hz, with 33 analyzed frequencies. The A-weighting was used for data.

In this case, two records were gathered about three minutes each, during different moments of the class. For both measures, a tripod was used. The height of the sound level meter was 1.50 cm from the floor. The sound level meters were placed together in the middle of the class.

### 3.2. Studied Population and Studied Area

In this subsection, we detail the studied population and the characteristics of the place where they do the classes.

We took our sample from the members of “Borumbaia drum school” in Valencia (teachers and students). Inclusion in the sample was based on voluntary participation, excluding people with a history of chronic ear diseases or surgeries (with the exception of transtympanic tubes during childhood). All patients were invited to participate in this study via email and group request in their weekly rehearsal. The main reason for not taking part was conflicting time schedules. We grouped a sample of 56 people. From 56 people, 43 of them completed the audiometries and questionnaires. From 43 people that form the studied population, 60.4% (26) are women and 39.6% (17) are males.

The room where the classes are performed and where the records of sound pressure levels were done is 14.785m^2^. It is rectangular shaped with dimensions of 9.075 m per 5.71 m and a height of 2.97 m. The room had been soundproofed to avoid the acoustic pollution to the surrounding buildings.

### 3.3. Equipment Used to Perform the Medical Test

In this subsection, the information of the medical instruments used in the test is shown. In addition, some information of the used questionnaire is detailed.

The subjects were studied using clinical examination, pure-tone and high-frequency audiometry, and a questionnaire. We asked about otological and head trauma background, number of years playing, weekly hours playing, and type of drum. Moreover, we ask them if they use or not protection measures during classes. Also, we recollected information about the presence of tinnitus, hyperacusis, or diplacusis. We tried to relate subjective hearing loss with the hearing thresholds found. We had questions about all their other noise exposure through their daily work, concerts, and other noisy hobbies.

Both tests and questionnaires were completed at the ear, nose, and throat department of the University General Hospital of Valencia. Clinical examination including otomicroscopy and cerumen was removed when needed to.

**Figure 2:**
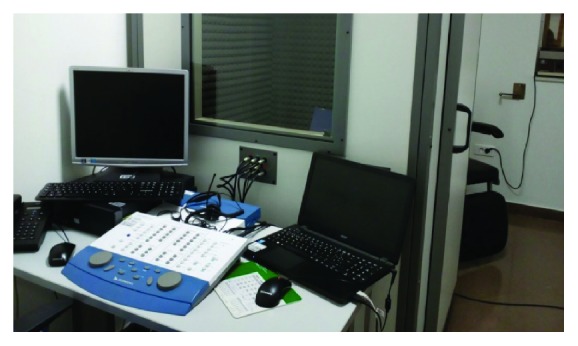
Employed audiometers.

We used a MADSEN Adstera2 audiometer (Otometrics) in a sound-attenuating room. Audiometry was bilaterally performed using the ascending method (ISO 8253-1, 2010) with a random first ear in the frequencies of 125, 250, 512, 1000, 2000, 4000, 6000, and 8000 Hz. The audiometer used can be seen in Figure 2.

### 3.4. Employed Protections by Musicians

In this subsection, we present the different musical instruments and protections used by the students.

**Figure 3:**
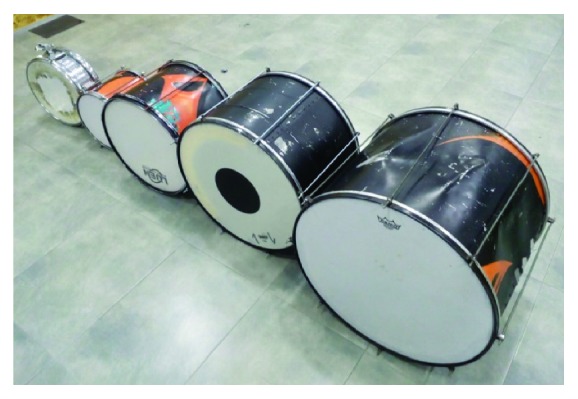
Instruments used at the school.

First, we show the different instruments. The instruments can be seen in Figure 3. In the school, there are four different instruments (from left to right): caixa, repinique, surdo (1), cutter surdo and surdo (2). The repinique is played by eight students, the surdo (1) and (2) by seven, the cutter surdo by 23, and the caixa by one. On the other hand, there are 6 students that have played different instruments.

Now, we detail the protection measures that the school offers to the teachers and students. They can use ear plugs and protection headphones. The earplugs are used by 19 students; the protection headphones by 16 and three of the students use both protections. Finally, there are five students that do not use any protection.

### 3.5. Hypotheses

In this subsection, we present the hypotheses that motivate our work. Our first hypothesis is that the SPL reached during a batucada class is higher than in other music classes. Because of this, the musicians and students can suffer from NIHL. This possible NIHL must be monitored in order to include this information in an e-health program to prevent the NIHL.

Our second hypothesis is that many musicians that are using the protection measures, especially the ones that are using the headphones may not use them properly. There are several reasons that can cause the protection headphones to not correctly protect the users. The main issue is the characteristics of the protection headphones; different headphones offer different acoustic isolation depending on their quality. If the headphones are not well adjusted to the skin of the users, its acoustic isolation decreases.

## 4. Assisted Protection Headphones

In this section, we show the developed assisted protection headphones. First, we describe the protection headphones. Then, its operation is described and its algorithm is shown.

**Figure 4:**
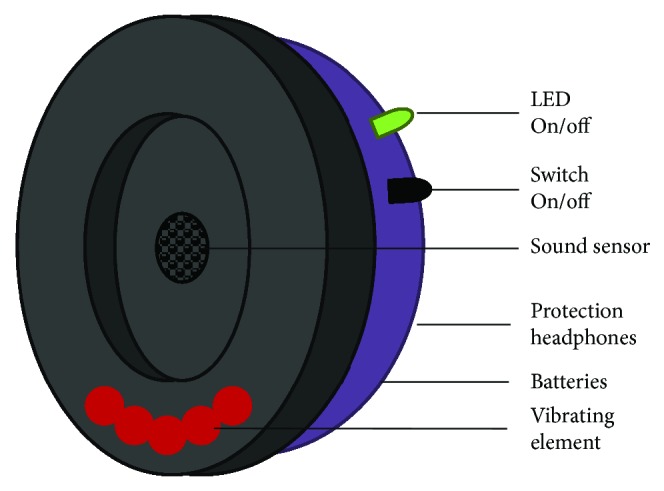
Assisted protection headphones with vibrating alarm.

We suspect that some musicians could be using the headphones incorrectly. For this reason, it is necessary, for musicians and other applications, to have protection headphones that help people to use them in a proper manner. We propose a new headphone that can inform the users if they are using it correctly. The assisted protection headphone can be seen in Figure 4. For this purpose, the sound sensor Arduino KY-038 Microphone sound sensor module will be used. The sensor will be placed in the inner part of both sides of the protection headphones. This sensor has a digital output when the sound pressure reaches an established threshold. When the threshold is exceeded, a vibration will be activated on the side where the sensor detects the sound. The vibration will alert the musician that the protection headphones are not properly adjusted. Then, musicians can adjust them in order to avoid further damages in the ears. Moreover, the sensor has an analog output that will allow gathering the sound pressure level in dB. The designed assisted protection has two main principal purposes. The first one is to assist the musicians to adjust the headphones properly. The second function is to monitor the exposition of each musician to the generated noise during the classes.

This assisted protection headphone will help to prevent NIHL by the use of sound sensors. They use an Arduino module to gather the data from the sensor and activate the vibrating element. The threshold value of the sound sensor will be established in 80 dB. The protection headphones must be activated with a switch button, and a green LED will indicate that it is operating. This system can assist different musicians, and other professionals, to use properly the protection headphones to avoid NIHL as a part of an e-health program for musicians.

The operation of the assisted headphones is described below. The proposed assisted protection headphones will be a part of a collaborative system. The proposed protection headphones will communicate with the headphones of the rest of the musicians. The objective is to differentiate two situations that can produce an increase in the SPL inside the headphones. Those situations are the inappropriate use of the protection headphones or an exceptional increase in the SPL outside of the protection headphones. The protection headphones attenuate part of the noise, in this case, 35%. If the external noise is too high, the protection headphones can be not enough. Those high noise levels can be caused by peaks in the music during the classes.

**Figure 5:**
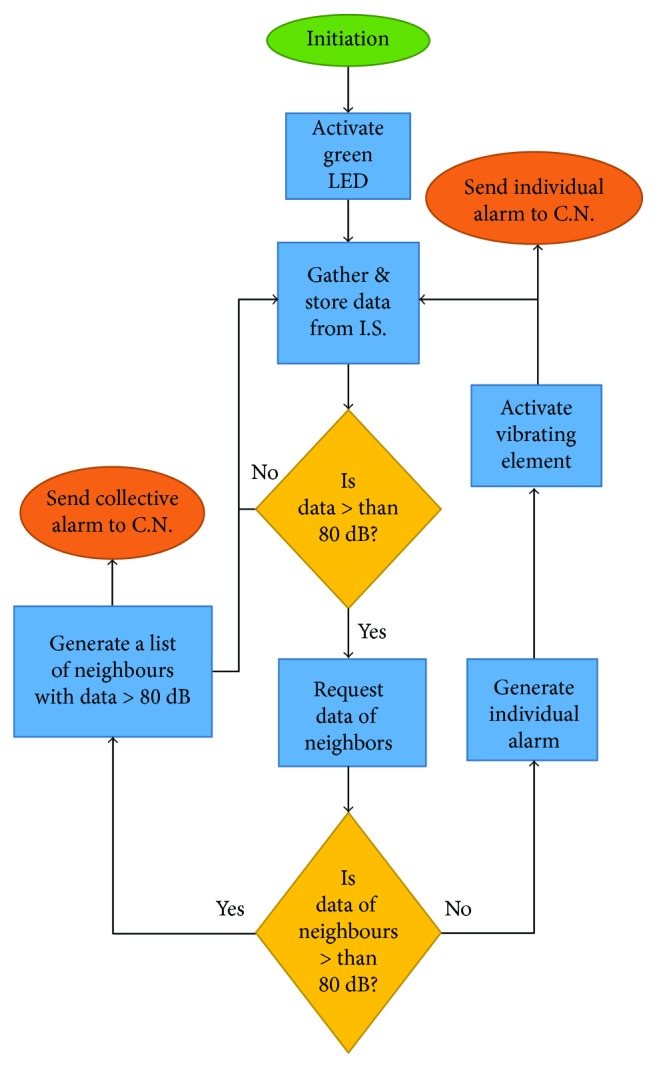
The algorithm of the assisted protection headphones.

To be able to differentiate between both situations, the following algorithm will be used, see Figure 5. First, the system turns on the green LED indicating that the system is on. Then, the headphones start to gather data of the internal sensor (IS) and store it in the internal memory of the Arduino. The next step is to compare the data with the established threshold, in this case, 85 dB. If the data is lower than the threshold, then the system continues gathering and storing data.

If the data is higher than the threshold, it is necessary to identify if this high value is caused by the incorrect use of the protection headphones or because there is a peak of noise. Then, the Arduino using its Wifi connection requests the data of its neighbors. If the data of the neighbors are below the threshold, then an individual alarm is generated. This individual alarm will produce vibrating elements that will turn on in order to advise the musician to properly adjust the protection headphones. Moreover, the alarm will trigger the node to send a message to the coordinator node (CN). If the data of the neighbors are also above the threshold, then a collective alarm is generated. This alarm will trigger the node to generate a list of the neighbors affected, and this information is sent to the CN. In this case, the vibrating element will not be triggered. The objective of generating a list of the affected musicians is to bring information in order to assess whether new protective measures are necessary for a specific group, by either their location or instrument. All this information is shown in real time in the smartphone of the teacher, which owns the CN. The teacher can assess their students and show them how to adjust the headphones according to the information of the generated individual alarms. If many collective alarms are generated they must consider using protection headphones with higher sound isolation properties.

**Figure 6:**
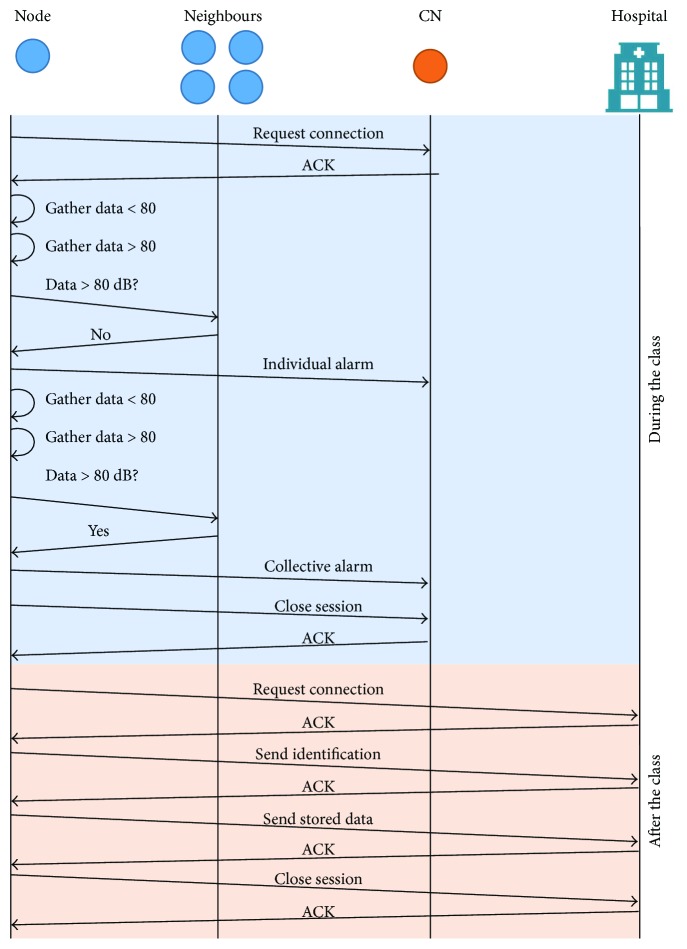
Message flow diagram during and after one class.

Moreover, the proposed system stores all the data gathered during the class. This information and the generated alarms are sent after the classes to the medical center where the students and professors are attending to monitor its NIHL (see Figure 6). This information will be assessed by the doctors in order to monitor the noise exposure and the treatment of the NIHL and as a part of an e-health program to prevent the NIHL.

## 5. Results

In this section, we present, on the one hand, the gathered data with the sound level meters during a regular session of batucada class. On the other hand, we present the data of the audiometries performed to the musicians. The statistical analysis shows the relationship between the obtained results in the audiometries and the protection measures employed or the musical instrument played. Moreover, the results of the test performed with the assisted protection headphones are presented.

### 5.1. Sound Records in the School

**Figure 7:**
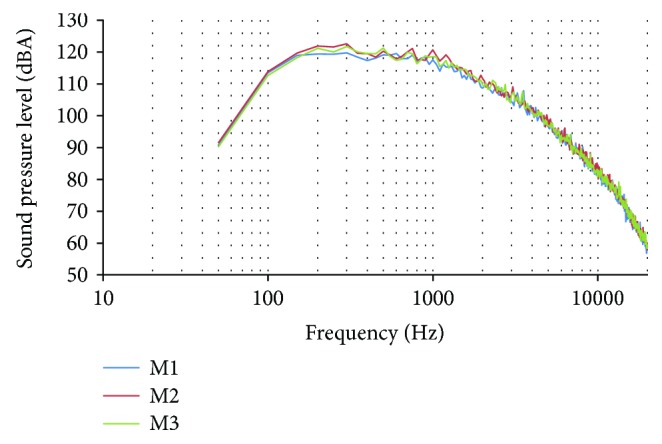
Maximum value of data gathered in the FFT analysis.

**Figure 8:**
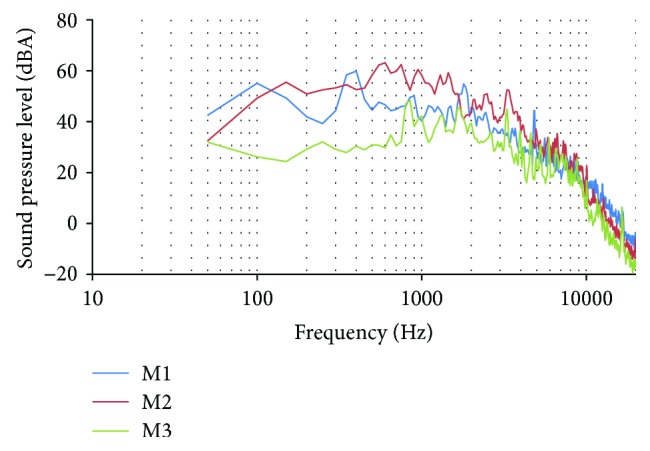
Mean value of data gathered in the FFT analysis.

In this subsection, the obtained data of the records with the sound level meter presented in the previous section is detailed. First, the results of the advanced FFT analysis are presented. The mean values for each frequency and the maximum value for each frequency in A-weighting is presented in Figures 7 and 8. We use the A-weighting as we saw this type of weighting in some assays about occupational noise-induced hearing loss [20]. The maximum SPL reached in the first record (M1) was 119.79 dB at 300 Hz (see Figure 7). The maximum values in the other records (M2) and (M3) were 122.60 dB at 300 Hz and 121.75 at 500 Hz. From the 400 analyzed frequencies, in the first record, 83 of them present SPL higher than 100 dB. On the second record, 89 frequencies have SPL higher than 100 dB. Finally, in the third record, 88 frequencies have SPL above 100 dB. The frequencies with higher SPL are between 100 and 4500 Hz. By the other side, the values of the mean SPL of each record are presented.

While in the maximum values, the profiles were similar in the three records, the profiles of the mean values are different in each record (see Figure 8). The maximum SPLs of the mean data are 59.93 dB at 400 Hz in M1, 63.17 dB at 600 Hz in M2, and 48.37 dB at 850 Hz in M3.

**Figure 9:**
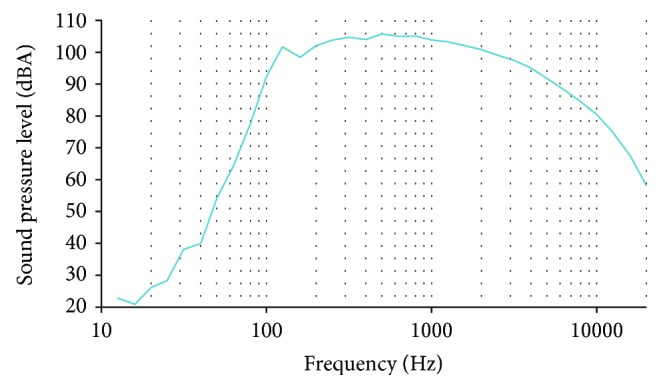
Data from LAeq gathered in the first frequency analysis.

**Figure 10:**
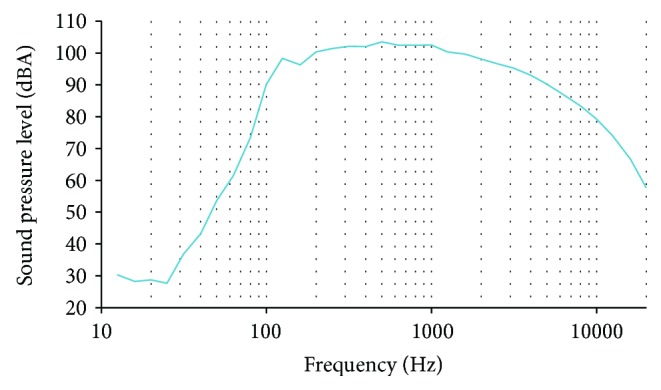
Data from LAeq gathered in the second frequency analysis.

Now, the results of the frequency analysis with A-weighting as the sound level in decibels equivalent to the total A-weighted sound energy measured over a stated period of time, LAeq, are shown in Figures 9 and 10. The mean data of both records are shown. This data, with the A-weighting, is similar to the human perception of sound. There is depletion in the frequencies lower than 1000 Hz. The maximum SPL reached in the first frequency analysis was 105.77 dB at 500 Hz. The maximum value in the second frequency analysis was 103.5 dB at 500 Hz. From the 33 analyzed frequencies, in both measures, 19 of them present SPL higher than 85 dB. SPLs higher than 85 dB are considered dangerous for the ear health. The frequencies with SPL higher than 85 dB are between 500 and 6300 Hz. Both records offer similar results.

### 5.2. Medical Test

**Figure 11:**
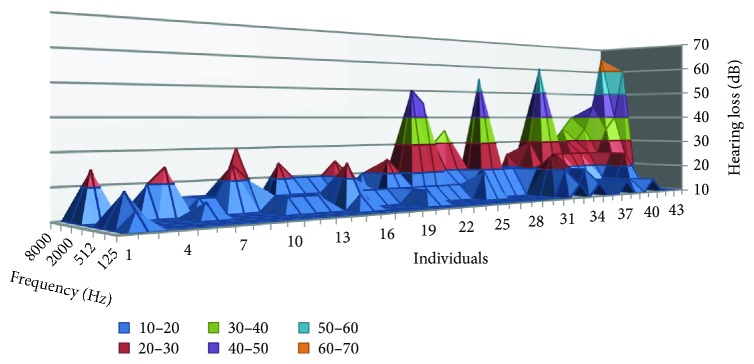
Results of the audiometry of each individual.

In this subsection, we show the obtained results of the audiometries performed to the musicians. First, we will show the NIHL at different frequencies. It can be seen in Figure 11. The test done was a bilateral pure-tone audiometry. Nevertheless, in Figure 11, we represent the worst result in both ears. The NIHL changes from one individual to another. At 125 Hz, the lowest analyzed frequency, from 43 studied individuals, only 4 present a hearing loss of 15 dB. The rest of them had a hearing loss of 10 dB. At 250 Hz, the maximum hearing loss was 20 dB, while the average hearing loss was 11 dB. The results at 512 Hz and 1000 Hz were similar, with a mean value of 12 dB and maximum hearing loss of 25 dB. The hearing loss was higher at frequencies 2000, 4000, and 8000 Hz. At those frequencies, the average hearing loss was 18, 25.5, and 18.4 dB, respectively, and the maximum individual response 60, 65, and 40 dB.

The hearing loss is defined when the hearing threshold showed in the audiometry is greater than 25 dB in two or more frequencies in the same ear or with one result greater than 30 dB. Furthermore, we have analyzed the NIHL of different subgroups.  Table 1 shows the individuals that present NIHL according to the exposure period. We can see that the groups of 2 years or less and 3 to 4 years of exposure present a similar % of people with NIHL. Nevertheless, in the group of 5 or more years of exposure, the % of people with NIHL doubles. In general terms, we can confirm that the exposure period has a relation with the NIHL in the case of batucada musicians.

The relationship between NIHL and the different instruments played is shown in Table 2. Musicians that played different instruments seem to have different percentages of NIHL in the group. People that play the cutter surdo seem to have less probability of NIHL (23.8%) than people that play repinique (50%). The surdo has less negative effects on hearing capability than repinique, but more than cutter surdo. The case of people that play caixa is not considered because only one person in our study group plays it.  Table 3 presents the relationship between NIHL and the use of different protections. We can see that people that use both protection measures do not present NIHL. The people that use earplugs have less prevalence of NIHL (26.3%) than people that use only the protection headphones (37.5%). Finally, in people that do not use any kind of protection measure, the % of NIHL is higher, 80%. It doubles the percentages of people that use the protection headphones and triples the one of those that uses earplugs.

Now, we are going to analyze the hearing loss at each frequency statistically and its relationship to the exposure period, instrument, and protection employed. For this analysis, we have used the statistical software Statgraphics Centurion [21]. The analysis of variance between groups (ANOVA) is employed. In this case, a multifactorial ANOVA is used to consider all the different factors (exposure period, instrument, and protection). Moreover, a new factor is included in this analysis. The new factor represents if in their daily life, they are exposed to other noise sources in their work or in other leisure activities. The ANOVA analyzes the mean and variance intra and inter groups in order to identify if the observed differences are because of the random data or because the groups are statistically different. This analysis is performed for each frequency.

**Figure 12:**
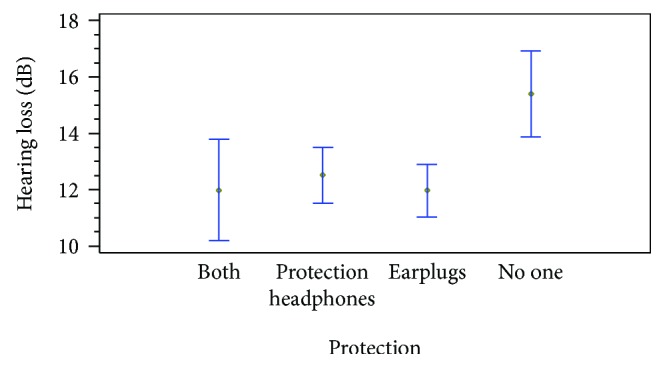
Hearing loss at 250 Hz with different protections.

At 125 Hz, the observed differences are related only to the played instrument. The people that play caixa, surdo, and cutter surdo present greater hearing loss than the others. At 250 Hz, the differences are explained because of the instrument and the protection. People that play caixa and surdo have a greater hearing loss at 250 Hz than the others. Moreover, the use of protections produces differences on hearing loss at 500 Hz.  Figure 12 shows the differences as the mean value in each group and the Fisher intervals. The people that do not use any protection have a greater hearing loss than the people that do. And this difference is statistically significant. At 500 Hz, the observed differences are explained by the instruments played. Again, the people that play caixa, cutter surdo, and repinique present greater hearing loss than the others. The results at 1000 Hz are also related to the instruments. The caixa and surdo are the ones related to greater hearing loss. At 2000 Hz, the hearing loss is mainly related to the other noisy activities.

**Figure 13:**
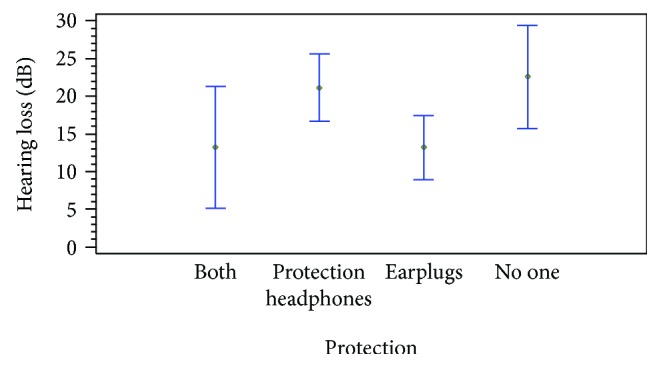
Hearing loss at 2000 Hz with different protections.

Moreover, the protection measures explain part of the difference. The people that do not use any protection present the worse hearing loss, followed by the ones that use the protection headphones. Again, the use of earplugs of both measures seems to be the best option to avoid the hearing loss. These differences at 2000 Hz, which can be seen in Figure 13, are not so big as the differences at 215 Hz. At 4000 Hz, the differences are caused by the other noisy activities developed by people. Because the small group of people evaluated and most of them are usually exposed to high levels of noise in their daily life, it is difficult to evaluate the effect that the batucada has in their NIHL. Finally, at 8000 Hz, no differences are observed.

### 5.3. Performance of the Assisted Protection Headphone

In this subsection, we are going to analyze the data gathered by two prototypes of assisted protection headphones and an external sensor during one batucada class.

**Figure 14:**
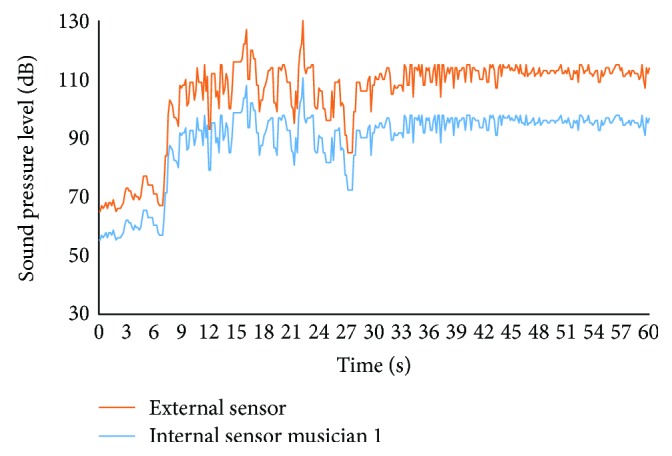
SPL of the external sensor and the internal sensor of musician 1.

**Figure 15:**
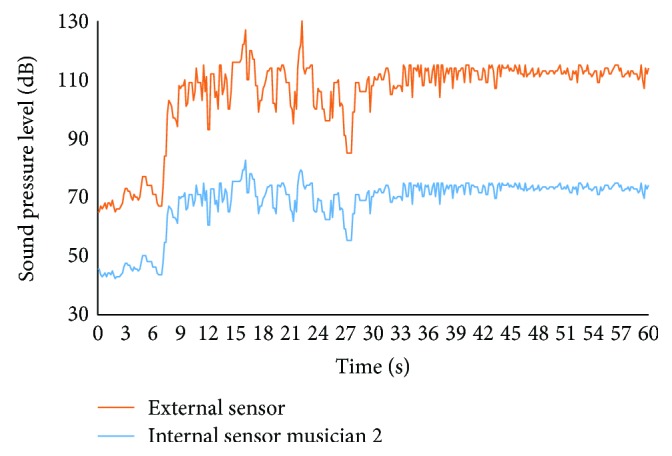
SPL of the external sensor and the internal sensor of musician 2.

Two musicians use the prototypes during one regular batucada class. While one of the musicians (musician 1) does not take care of adjusting the protection headphones, the other one takes care and adjusts the headphones properly (musician 2). Moreover, an external sensor is placed between the musicians in order to have a reference. The system was used during the first 60 s of the class. In Figure 14, the data gathered by the external sensor and the sensor of musician 1 are presented. The data that belongs to the sensors of musician 2 is shown in Figure 15. The difference between the external sensor and the sensors of the musicians is related to the isolating proprieties of the protection headphones. The decrease of the SPL is higher in musician 2 than in musician 1. The maximum SPL gathered by the external sensor is 127 dB. In this moment, the data gathered by the sensor of musician 1 is 108 dB and the data from musician 2 is 83 dB. The mean SPLs gathered by the external sensor, sensor from musician 1 and musician 2, are 104.9 dB, 89.2 dB, and 68.2 dB.

**Figure 16:**
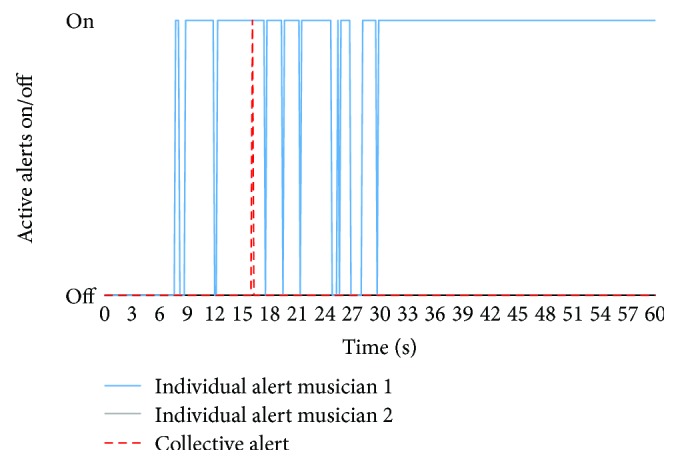
Generated individual and collective alarms during the test.

The generated alarms during this class can be seen in Figure 16. During the first seconds, when the batucada starts to play, no alarms are generated. Nevertheless, at the second 7, the alarm of musician 1 turns on for the first time. The individual alarm of musician 1 turns on 10 times during 60 s of the test. On the other hand, the individual alarm of musician 2 remains turned off during the entire test. At the second 16, coinciding with the maximum peak, the collective alarm turns on.

### 5.4. Discussion

In this subsection, we will analyze the observed differences in the audiometries with the obtained data of the sound meters.

From the obtained data, we can see that SPL higher than 120 is reached during the batucada class and these levels are considered as a dangerous SPL and can cause NHIL. This confirms our first hypothesis that the SPL reached in the batucadas is dangerous and some protection must be used to prevent NIHL. The SPL gathered during a batucada class are similar to the ones detected in rock bands [4, 5].

According to the measures taken in the classroom, the peak frequencies are at 500 Hz. The frequencies that present sound pressure level considered as dangerous are between 500 and 6300 Hz. We detected that during different moments of the class, the results were different. For this reason, it is possible to affirm that different instruments can reach different peak frequencies and produce different alterations.

The statistical analyses of the medical test show that there is a hearing loss in many members of the studied musicians. From the 43 studied individuals, 15 present NIHL. The NIHL is different in people with different exposure period, people that play different instruments, and people that use different protection measures. This data is confirmed by the performed ANOVAs per frequencies. At 250 Hz, the hearing loss is related to the protection measures and the played instrument. Similar results are found at 512, 1000, and 2000 Hz. No data show that the hearing loss is related to the exposure period. This data confirms our second hypothesis; the musicians are suffering from NIHL even when they are using protection headphones.

We can relate the frequencies where we detected SPL (greater than 100 dB) with the frequencies where musician groups have hearing loss. The frequency analysis has shown greater SPL between 125 Hz and 2000 Hz. Statistical analysis in the audiometry has shown a significant difference between different groups between 250 Hz and 2000 Hz.

The developed assisted headphones show how the internal SPL when the headphones are not well adjusted is higher. In the test, the musicians that do not adjust properly the headphones reach peak internal SPL higher than 100 dB.

## 6. Conclusion

The NIHL is a common problem in different sectors of the human population. It is well studied in different professional sectors. However, it is less studied in the leisure environment. In this paper, we developed different tests with musicians of the “Borumbaia drum school” in Valencia. They generally play batucada. The results show that, during a regular class, the peak values are 122.60 dB at 300 Hz and 121.75 at 500 Hz. From the 400 analyzed frequencies, 88 of them have SPL higher than 100 dB. The maximum SPL is found from 125 Hz to 2250 Hz. The audiometries show that 35% of the studied musicians present NIHL. Some of the musicians that use protection headphones as protection measure present NIHL. We suspect that some musicians do not use the protections properly. For this reason, an assisted protection headphone and its operation protocols are designed to ensure the correct adjustment of protection headphones as a part of the e-health program to diminish the effects of high PSL on HINL. A simple test developed during a regular class shows how when a musician does not adjust properly the headphones the peaks of SPL reached in the internal part of the headphones are higher than 100 dB, while if the headphones are properly adjusted they are reduced to 83 dB.

Our future work will be focused on studying the sound pressure levels reached by each instrument individually. The data will be gathered by the same methodology employed in this paper but using the A-weighting and C-weighting. Moreover, we will include a higher population with and without high levels of noise in their daily lives. This is aimed to define the observed differences in NIHL between musicians that play different instruments. Moreover, we pretend to evaluate the use of assisted protection headphones in other cases. We will also include indoor self-location systems in order to detect the placement of each headphone [22, 23].

## Figures and Tables

**Table 1:** Relation between NIHL and exposure period.

Exposure years	People with NIHL	People without NIHL	% of people with NIHL
2 or less	1	3	25
3 or 4	7	18	28
5 or more	7	7	50

**Table 2:** Relation between NIHL and played instrument.

Instrument	People with NIHL	People without NIHL	% of people with NIHL
Cutter surdo	5	16	23.8
Caixa	1	0	100
Repinique	4	4	50
Surdo	3	4	42.9
Many	2	4	33.3

**Table 3:** Relation between NIHL and protections used.

Protection	People with NIHL	People without NIHL	% of people with NIHL
Both	0	3	0
Protection headphones	6	10	37.5
Earplugs	5	14	26.3
No one	4	1	80
